# Supplementary material for: Measurement of fasted state gastric antral motility before and after a standard bioavailability and bioequivalence 240 mL drink of water: Validation of MRI method against concomitant perfused manometry in healthy participants
Source: PLoS One. 2020 Nov 11;15(11):e0241441. doi: 10.1371/journal.pone.0241441 (PMC7657519; doi:10.1371/journal.pone.0241441)
Supplement: Attachment — Submitted filename: Response to Reviewers.docx [file pone.0241441.s001.docx]

**PONE-D-20-12517 REVISION R1: Measurement of fasted state gastric antral motility before and after a standard bioavailability and bioequivalence 240 mL drink of water: validation of MRI method against concomitant perfused manometry in healthy participants**

We are grateful for the opportunity to revise our manuscript and we thank the Academic Editor and the Reviewers for their constructive comments. We believe that this process has considerably improved the paper.

We address first the Journal/Editorial points raised and secondly the Reviewers’ comments in the point-by-point the detailed answer below. All changes made to the main manuscript are tracked in the marked-up Word version enclosed. Some Editorial points are also addressed in the enclosed Cover Letter as requested.

**Editorial and Journal points**

*If you would like to make changes to your financial disclosure, please include your updated statement in your cover letter. Guidelines for resubmitting your figure files are available below the reviewer comments at the end of this letter.*

**Answer**

Thank you, we have updated our disclosures as detailed below and in the Cover Letter. We have also resubmitted the figures after passing them through the PACE engine as required.

*If applicable, we recommend that you deposit your laboratory protocols in protocols.io to enhance the reproducibility of your results.*

**Answer**

The study protocol is publicly available on Clinicaltrials.gov (NCT03191045) as already referenced in the manuscript. We do not have extensive protocols to deposit in protocols.io.

*1. Please ensure that your manuscript meets PLOS ONE's style requirements, including those for file naming. The PLOS ONE style templates can be found at*

[*https://journals.plos.org/plosone/s/file?id=wjVg/PLOSOne_formatting_sample_main_body.pdf*](https://journals.plos.org/plosone/s/file?id=wjVg/PLOSOne_formatting_sample_main_body.pdf) *and*

[*https://journals.plos.org/plosone/s/file?id=ba62/PLOSOne_formatting_sample_title_authors_affiliations.pdf*](https://journals.plos.org/plosone/s/file?id=ba62/PLOSOne_formatting_sample_title_authors_affiliations.pdf)

**Answer**

We ahve checked our manuscript against the templates adn checked the file naming, the format corresponds to the requirements

*2. a) Please amend your current ethics statement to include the full name of the ethics committee/institutional review board(s) that approved your specific study.*

*2. b) Once you have amended this/these statement(s) in the Methods section of the manuscript, please add the same text to the “Ethics Statement” field of the submission form (via “Edit Submission”).*

**Answer**

Thank you, the full name of the University of Nottingham Faculty of Medicine and Health Sciences Research Ethics Committee is now included at line 141. The full name of the FDA ethics committee already appeared. The statement now reads “The study was conducted at the Sir Peter Mansfield Imaging Centre (SPMIC) at the University of Nottingham. The study was approved by the University of Nottingham Faculty of Medicine and Health Sciences Research Ethics Committee (A14112016) and by the US Food and Drug Administration Research Involving Human Participants Committee (16-073D). All participants gave written, informed consent before joining the study.” and it has been added to the submission form on Editorial Manager.

*3. We note that you have indicated that data from this study are available upon request. PLOS only allows data to be available upon request if there are legal or ethical restrictions on sharing data publicly. We will update your Data Availability statement on your behalf to reflect the information you provide.*

**Answer**

Thank you, we had misunderstood this point. We have now saved the anonymized individual data and the time courses in an Excel file which we have uploaded to our Institution’s public repository, the Nottingham Research Data Management Repository (<https://rdmc.nottingham.ac.uk/> ).

The repository satisfies all major funders’ requirements for data access. The data file is now publicly available with this permanent DOI **10.17639/nott.7068** and this is noted also in the Cover Letter

*4. Thank you for stating the following in the Financial Disclosure section:*

*' The authors GEA, GLA and LM received for this work a grant from the U.S. Food and Drug Administration (FDA),* [*https://www.fda.gov/home*](https://www.fda.gov/home)*, Contract HHSF223201510157C. The funders had no role in study design, data collection and analysis, decision to publish, or preparation of the manuscript. This article therefore reflects the views of the authors and should not be construed to represent FDA’s views or policies.'*

*We note that one or more of the authors are employed by a commercial company: Motilent Ltd, and Capsugel*

*a. Please provide an amended Funding Statement declaring these commercial affiliations, as well as a statement regarding the Role of Funders in your study. If the funding organization did not play a role in the study design, data collection and analysis, decision to publish, or preparation of the manuscript and only provided financial support in the form of authors' salaries and/or research materials, please review your statements relating to the author contributions, and ensure you have specifically and accurately indicated the role(s) that these authors had in your study. You can update author roles in the Author Contributions section of the online submission form.*

*Please also include the following statement within your amended Funding Statement.*

*“The funder provided support in the form of salaries for authors [insert relevant initials], but did not have any additional role in the study design, data collection and analysis, decision to publish, or preparation of the manuscript. The specific roles of these authors are articulated in the ‘author contributions’ section.”*

*If your commercial affiliation did play a role in your study, please state and explain this role within your updated Funding Statement.*

**Answer**

Agreed, at point a. we have now included in the revised funding statement that co-author DMM is currently employed by company Capsugel and that co-author AM is CEO of Motilent Ltd and have specified in more detail their role in this work, also updated AM’s role more precisely in the ‘Author Contribution’ section.

We also added the suggested statement at point on the role of the funder.

**REVISED FUNDING STATEMENT:** ' The authors GEA, GLA and LM received for this work a grant from the U.S. Food and Drug Administration (FDA), https://www.fda.gov/home, Contract HHSF223201510157C. The author DMM is currently employed by company Capsugel and author AM is CEO of Motilent Ltd. The funders and company Capsugel had no role in study design, data collection and analysis, decision to publish, or preparation of the manuscript. Company Motilent provided the data analysis software as part of a subcontract on the research grant and had no role in study design, data collection and decision to publish. AM helped to with set up of the image analysis software and image data registration and with preparation of the final manuscript but had no role in the actual study data analysis. The funder provided support in the form of full salaries for authors KH, NA and fractional salary cost for CLH, JW GEA, GLA and LM, but did not have any additional role in the study design, data collection and analysis, decision to publish, or preparation of the manuscript. The specific roles of these authors are articulated in the ‘author contributions’ section.This article therefore reflects the views of the authors and should not be construed to represent FDA’s views or policies.'

*b. Please also provide an updated Competing Interests Statement declaring these commercial affiliations along with any other relevant declarations relating to employment, consultancy, patents, products in development, or marketed products, etc.*

*Within your Competing Interests Statement, please confirm that these commercial affiliations do not alter your adherence to all PLOS ONE policies on sharing data and materials by including the following statement: "This does not alter our adherence to  PLOS ONE policies on sharing data and materials.” (as detailed online in our guide for authors* [*http://journals.plos.org/plosone/s/competing-interests*](about:blank)*) . If this adherence statement is not accurate and  there are restrictions on sharing of data and/or materials, please state these.*

*Please note that we cannot proceed with consideration of your article until this information has been declared.*

*Please know it is PLOS ONE policy for corresponding authors to declare, on behalf of all authors, all potential competing interests for the purposes of transparency. PLOS defines a competing interest as anything that interferes with, or could reasonably be perceived as interfering with, the full and objective presentation, peer review, editorial decision-making, or publication of research or non-research articles submitted to one of the journals. Competing interests can be financial or non-financial, professional, or personal. Competing interests can arise in relationship to an organization or another person. Please follow this link to our website for more details on competing interests:* [*http://journals.plos.org/plosone/s/competing-interests*](about:blank)

**Answer**

Agreed. We have revised our Competing Interest statement accordingly as show below.

**REVISED COMPETING INTEREST STATEMENT:** I have read the journal's policy and the authors of this manuscript have the following competing interests: Authors GEA, GLA and LM were recipient of research grant # HHSF223201510157C by the U.S. Food and Drug Administration (FDA). This paid for research salaries and consumables for this work. The author AM is the CEO of Motilent Limited and the author DMM is an employee of Capsugel. This does not alter our adherence to PLOS ONE policies on sharing data and materials.

*c. Please include both an updated Funding Statement and Competing Interests Statement in your cover letter. We will change the online submission form on your behalf.*

**Answer**

This has been included in our cover letter.

**Additional changes**

We have corrected affiliation 9 to the ‘Department of Statistics’ not Public Health as incorrectly stated

**Response to Reviewer 1 Comments**

*“There is not much statistical content, other than some Pearson correlations and a mixed model. So I would argue about the term "spatio-temporal", as there is no multidimensional spatial/time series analyses, but only a simple repeated measures.”*

**Answer**

Thank you for this comment. In the statistics literature, a spatio-temporal analysis models data both spatially and temporally. This study collected spatio-temporal data, but the planned analysis produced one number summaries of motility per subject and sampling period, thus collapsing the spatio-temporal data to two AUC statistics and losing some account of spatio-temporal qualities. As such we agree with the suggestion and have removed the “spatio-temporal” term throughout the manuscript unless specific, like for example in the case of the initial MRI raw data (STMM, reference 31) analysis technique which is published with the full spatio-temporal mapping features.

*“Sample size is small and seems to have been chosen at random, as there is no power computation.”*

**Answer**

Thank you for querying this, we agree a comment to this effect was missing. We have now included the following at Lines 255-266 (numbering from the clean version with no tracking): “The study design included power analysis considerations. Briefly, for correlation analyses involving data that are summarized to one number per subject, the standard error for the estimated correlation coefficient over 15 subjects is 0.28. However, our main analyses use time-resolved data with an overall sample size of 421 values over the 15 subjects. Using a Kronecker sum model to capture within-modality autocorrelation (for MRI or manometry) and between-modality correlation (the parameter of interest), much smaller standard errors are obtained. For example, if the within-modality autocorrelation is 0.5, the between-modality correlation coefficient is estimated with a standard error of 0.049. Thus, the confidence intervals for the correlation parameter of interest will be reported as the estimate +/- 0.1, giving us excellent power to resolve weak from strong correlation. The standard error depends on the within-modality autocorrelation, which was not known at the time of study design. The actual data obtained showed high precision of the estimated correlation coefficient, indicating that the a priori study design was successful."

*“P-values on correlations are really meaningless, because they test 0 correlation against "some" correlation. In this case, a significant p-value doesn't necessarily mean the correlations are strong.”*

**Answer**

Thank you for this, we agree with this point, we removed the p values on the correlations throughout.

We also noted we had quoted R2 values which are now replaced by correlation r values

*“The regression models need a careful residual analysis.”*

**Answer**

Agreed, a careful residuals analysis was performed but not included in the manuscript. The residual analysis led us to conclude that the non-linear model fit the data better than the linear model.

*“I think there could have been a lot more done with these data, but maybe the sample size is too small.”*

**Answer**

The additional analysis was beyond the scope of the manuscript and what had been planned at funding stage. The possibility to use these data towards optimization and validation of *in vitro* and *in silico* predictive biopharmaceutic models will be explored. The sample size has now been addressed in answer to another point raised.

**Response to Reviewer 2 Comments**

*“This manuscript reports on the use of non-invasive dynamic magnetic resonance imaging coupled with semi-automated spatio-temporal data analysis for measuring fasted-state gastric motility and validation of such a method vs. intraluminal pressure recording by means of perfused manometry technique.
 Human gastrointestinal motility patterns are of utmost importance to oral drug delivery. The investigated RMI-based method would represent a valuable advancement in the relevant study and allow to gain better insight into the impact of many variables. The work is very interesting, broad in scope, clearly exposed and in-depth discussed.”*

**Answer**

Thank you for the positive appraisal of our work.

*“The title is rather long: is the word “imaging” actually needed when the acronym RMI already contains it?”*

**Answer**

Agreed, we deleted the word ‘imaging’ and in response to Reviewer 1 we also deleted the ‘spatio-temporal mapping’ words thus making the title shorter.

*“Lines 113-114: as the two methods correlated with each other, “both” sounds odd and can be deleted.”*

**Answer**

Agreed, this was corrected as suggested.

*“Lines 123-124: “Both studies did not use semi-automated analysis methods” may better read “Neither of the studies used…”.”*

**Answer**

Agreed, we rephrased this sentence as suggested.

*“Line 125: please delete the comma after “making”.”*

**Answer**

Agreed, the comma was deleted.

*“Lines 127-128: “more operator independent” may be changed into “less operator-dependent” (this also applies to line 400).”*

**Answer**

Agreed, both sentences were adjusted as suggested.

*“Line 143: “Exclusion criteria included..” sounds a bit strange. “Exclusion criteria were”? Or “encompassed”?”*

**Answer**

Agreed, we used the word ‘encompassed’ instead of ‘included’.

*“Fig. 2: the caption should be changed into a more concise form taking account of the detailed description and explanation already provided in the text. When the content of the image and the meaning of green and yellow lines are indicated, it will be enough.”*

**Answer**

Agreed, we revised the figure legend of Figure 2 shortening it as follows: “**Fig 2. Image analysis.** Example of MRI data analysis. The stomach wall boundaries and the axis of the stomach are shown in green. The yellow lines represent the lumen diameter perpendicular to the stomach axis. AC, ascending colon; TC, transverse colon; GB, gall bladder.”

*“Line 257: “Each subject participated in either one or two intubation studies” seems to clash with the Methods section reporting that the volunteers “…took part in two identical fasted state MRI study visits” (lines 149-150) and “This was a single-centre, open-label design study that consisted of two separate identical study days...” (lines 154-155).”*

**Answer**

Agreed, thank you, we deleted the sentence on line 257.

*“Lines 297-298: please check and rephrase “The correlation for these data shows a significant positive correlation …”.”*

**Answer**

Agreed, we rephrased the sentence: “A positive, significant correlation was observed between the two techniques (R² = 0.7134, p < 0.001).“

*“Fig. 6: please check the legend as colors are not visible in the figure whereas dotted/solid lines and different symbols are used as distinguishing features for the 2 curves.”*

**Answer**

Thank you for spotting this, this was a typo that slipped in from an older draft version, we now refer to the curves as solid and dotted lines.

*“Lines 337-340: please check and rephrase” The fasting state and the use of a 240 mL dose of water as a challenge were specifically chosen because of their relevance to the bioavailability/bioequivalence (BA/BE) studies in the fasted state”, as it is implied that fasting volunteers take part in fasted-state bioavailability/bioequivalence studies. It seems that only 240 ml of water was chosen because of relevance to those studies.”*

**Answer**

Thank you we checked the phrase and it is correct. During the clinical trials of oral drug product development, it is common practice (and FDA guidelines which we refer to) to ask the participants to fast overnight (hence the fasted state) and to take the drug product of interest drinking 240 mL of water. Our protocol copied this with the difference that our participant were not asked to ingest a drug product.

This was not clearly stated so we have added now at lines Therefore, we added the following sentences at Lines 354-357: “However, it should be noted that the participants here did not actually ingest a drug product. The recommended 240 mL of water was applied as this volume is also used in BA/BE studies during the clinical phase of drug product development.”

*“Lines 354-355: “When the stomach is active, this will cause the manometry AUC to increase more quickly than the MRI AUC….” does not appear strictly related to the previously-mentioned circumstance. Could this be an additional factor differing between the two methods?”*

**Answer**

We do see that the manometry AUC can increase more than the MRI AUC, but only seen for high pressure (mmHg) values. At a certain point, when the stomach completely contracts, no differences in MRI AUC will be observed but still some increase in manometry AUC can be noted as the pressure will increase (increasing force of the stomach towards the water-perfused electrodes), but distance will not change anymore. That is the reason why we see this non-linear correlation between both techniques at higher AUC values. The word ‘quickly’ however was vague and it has now been removed.

*“Line 364: should “complimentary” read “complementary”?”*

**Answer**

Thank you the spelling was corrected.

*“Line 382: “a relative short time window” (or without “a”).”*

**Answer**

Agreed we deleted “a”.

*“Line 393-394: “an oral administered” should read “an orally administered””*

**Answer**

Thank you, this was adjusted.

*“Line 396: as no statistical optimization study is concerned, “optimized” could be replaced by a different verb. The same applies to lines 418 and 438 (“optimization”).”*

**Answer**

Thank you for this comment; we deleted the word ‘optimized’ and replaced it with ‘developed’ at Line 414, also deleted ‘optimization’ at old numbering Lines 418 and 436

*“Lines 315-319: this concept is already mentioned at lines 390-394. Please revise or synthesize the two similar passages in order to avoid repetitions.”*

**Answer**

Having looked at this we think the Reviewer may have meant that lines 415-419 were repeating the concept at lines 390-394 about non-invasiveness, other parameters measurable and use for drug dissolution studies (315-319 are in the results relating to different things). We agree this was repeating itself a bit and we have now removed the lines at the second occurrence “The MRI technique is able to monitor different GI variables simultaneously (i.e. GI motility, gastric emptying and residual fluid volumes) which is of great interest for pharmaceutical purposes in order to study the impact of GI variables on drug and formulation behaviour.”

*“Lines 431: “gastric motility” can be deleted as it is specified again at line 432, same sentence.”*

**Answer**

Agreed, “Gastric motility” was deleted at the first occurrence.

**Response to Reviewer 3 Comments**

*“The manuscript is interesting and well written. It is not the first time that MRI has been shown to be a valid method for the measurement of gastric emptying, secretion, motility and intragastric distribution of gastric contents. This study, however, adds value to the use of MRI that assess gastric motility by using a new spatio-temporal MRI mapping technique. Results of this study validate this new MRI assessment by comparing its measurements to simultaneous water-perfusion manometry after ingestion of 240 ml of water with the aim to study pharmacokinetics in humans.”*

**Answer**

Thank you for this positive appraisal.

*“However, I have some remarks.
1. The aim of the study is a bit confusing. In the introduction, justification for this new technique is done by explaining the lack of methods for the assessment of predictive drug dissolution models in humans. This aim seems to change in the discussion and it highlights the use of this new method mainly and rather as a gastric motility assessment technique. As the initial aim was to validate this new technique as a method to study pharmacokinetics and therefore dissolution of drugs, this should be further addressed more clearly in the discussion section. Additionally, a proof of concept study should be suggested as a next step to investigate the actual assessment of pharmacokinetics.”*

**Answer**

Correct statement. During the past years, there was specific focus on validating existing in vitro and in silico models for their capacity to predict the in vivo performance of an orally administered drug product to humans. Numerous projects (e.g., OrBiTo, PEARRL, UNGAP) were designed to explore the predictive performance of existing in vitro and in silico models. However, there is still a general scarcity of data on gastric motility to help us understand the impact of gastric motility on an orally administered dosage form. With the MRI techniques one can look at undisturbed (no intubation) contractility. As a recent study by Hens et al. successfully implemented MRI-derived dynamic fluid volumes in commercially-available computational software (e.g., GastroPlus), we will be able now to do the same for motility patterns and investigate how this will impact formulation disintegration for instance.

We agree with you that something was missing in the Discussion section. We added there at Lines XXX-YYY a sentence to better link this study with pharmacokinetic studies: “With respect to future uses of the MRI technique, a pharmacokinetic study in combination with assessment of motility could provide unprecedented insights on inter-subject variability in systemic outcome of a drug, as the underlying motility patterns are likely one dominant cause of known inter-subject differences in formulation disintegration and, subsequently, dissolution and absorption of the drug. These type of studies are the next logical step to further our understanding of to investigate the actual assessment of pharmacokinetics variables”

*“I do not agree with some section in the discussion addressing focus to the validity and accuracy of this new method for the assessment of gastric motility with a broader spectrum, including assessment of general GI function in GI disorder or the impact of drugs to GI motility. The ingestion of 240 ml of water studied in the present study is not enough to make such a statement. Therefore, careful should be taken in the discussion and it should be indicated for such cases further validation studies should be needed.”*

**Answer**

Agreed, the last 5 lines of the Discussion were speculative as to the validity and accuracy of these methods beyond the data presented and were removed. The corresponding statements in the Abstract and in Conclusions have been removed accordingly.

*“2. Where are the sensors of the water-perfusion manometry located? Is this antral and duodenal assessment? Please, specify in methods.”*

**Answer**

Good point, agreed, we have clarified at Lines 180-182 that “The perfused manometry ports tend to record primarily antral contractions whilst more proximal sensors in the wider fundal region do not record contractions well, as such the assessment here was focused on the antral region of the stomach.”

*“ 3. Following up the previous comment, if sensors are only located in the antral section of the stomach, and thus assessing antral contractions, this should also be specified throughout the entire manuscript: validation of this technique is for ANTRAL motility assessment and not general gastric motility assessment.”*

**Answer**

Agreed, we have specified ‘antral motility’ in the title and several times throughout the manuscript.

*“4. What was the drinking speed of the volunteers? Do they had to drink all water within 5 minutes or less? This was done in the MRI in supine position. How was this managed? Specify in methods.”*

**Answer**

Agreed, we now specify at Lines 206-208 that the participants drank while they were sitting up on the scanner bed and that they were not instructed to drink the water at a given speed, but all of the participants drank the glass of water quickly, within approximately one minute.

*“5. In the methods it is discussed a “the manometer was connected to the MRI compatible water-perfused manometry system”. What does this means? That the manometry device is all-plastic? Is this the research’s group own developed system or was this bought from a company? In this last case, from which company?”*

**Answer**

Agreed, some detail was missing, thank you for pointing this out, we now added at lines 183-187 the system manufacturer (the Biomedical Engineering Department at The Royal Melbourne Hospital in Australia) and describe that the transducers connecting to the catheter were secured to a MRI-compatible trolley placed next to the scanner bed. Electric cables and pipes went through a wave guide opening and connected the transducers box to the electronics and pressure gas cylinder placed outside the scanner room.

*“6. Include reference and explain possible limitation regarding supine positioning and its effect on GI motility (Might have an effect on the gastric emptying rate):* *Treier R, Steingoetter A, Weishaupt D, Fried M, Boesiger P, Schwizer W. Gastroenterology 2003; Gastric motor function and emptying in the right decubitus and seated body position as assessed by magnetic resonance imaging”*

**Answer**

Thank you for raising this point. We added a comment with reference to that paper and also to the other paper from the same group whereby they put people upside down in a vertical scanner at lines 438-443 with the two references.

*“I think in the figures 4 and 5 the “AUC” for MRI is missing.”*

**Answer**

Thank you for spotting this, this was added to both figures.

*“8. Figure 6. Can you include in the figure the moment the water was ingested?*

**Answer**

Agreed, the drink ingestion time has been indicated with an arrow and text in Fig 6.

*Why is there a gap between -15 and 0?*

**Answer**

As described at Lines 207-208 the participants were allowed a rest outside the scanner room before the drink part of the study. To allow them to move we disconnected the perfused manometry system (Line 208). None of the participants wanted a long break, they took only advantage of the rest time to stretch their legs and visit the toilet before continuing and the average time including reconnection was 15 minutes corresponding to the gap. We have clarified the timing at liens 208-211

*Why is the pressure so low at time 0?”*

**Answer**

We have a comment to this effect at Lines 365-368 of the Discussion where we indicate that is likely to reflect the inability of the perfused manometry to measure non-occlusive events when a free fluid is present to redistribute the pressure changes instantaneously.

*Figures Check*

*While revising your submission, please upload your figure files to the Preflight Analysis and Conversion Engine (PACE) digital diagnostic tool,* [*https://pacev2.apexcovantage.com/*](https://pacev2.apexcovantage.com/)*. PACE helps ensure that figures meet PLOS requirements. To use PACE, you must first register as a user. Registration is free. Then, login and navigate to the UPLOAD tab, where you will find detailed instructions on how to use the tool. If you encounter any issues or have any questions when using PACE, please email PLOS at* [*figures@plos.org*](mailto:figures@plos.org)*. Please note that Supporting Information files do not need this step.*

**Answer**

Agreed, we have passed the figures through the PACE engine and uploaded the PACE Corrected files
